# Parents’ Cigarette Purchases During COVID-19: Evidence From NielsenIQ Consumer Panel Data

**DOI:** 10.1016/j.amepre.2025.107980

**Published:** 2025-07-15

**Authors:** Lauren E. Jones, Nahae Kang, Tansel Yilmazer

**Affiliations:** 1 John Glenn College of Public Affairs, The Ohio State University, Columbus, Ohio;; 2 Consumer Sciences, Department of Human Sciences, The Ohio State University, Columbus, Ohio

## Abstract

**Introduction:**

Although national sales of cigarettes increased by 14% during the first months of COVID-19, which households bought more cigarettes is not yet known. This study illustrates which households bought more cigarettes during COVID-19 and examines whether changes were related to parental status and race/ethnicity.

**Methods:**

Using 2019 and 2020 NielsenIQ consumer panel data on U.S. households, a cohort study using fixed-effects regressions was conducted to test for changes within households in the probability of purchasing any cigarettes and the number of cigarettes before and after the onset of the pandemic. Analyses were conducted to examine whether Black and Hispanic parents changed their purchase behavior differently compared with comparison households. Analyses were conducted in 2024.

**Results:**

After COVID-19, the average household was not more likely to buy cigarettes, and Hispanic parents were about 10% less likely to buy. The average smoking household bought about 11 more cigarettes per week. Black parents who smoked bought about 25 additional cigarettes per week.

**Conclusions:**

There was minimal take-up of smoking during COVID-19, but those who already smoked bought an extra half a pack per week. Black parents who smoked nearly doubled the number of weekly cigarettes they bought. There is a risk of increased secondhand smoke exposure for Black children during COVID-19, potentially worsening racial health gaps.

## INTRODUCTION

The onset of the coronavirus disease 2019 (COVID-19) pandemic refocused attention on the perils of smoking. Smoking was identified as a risk factor for severe COVID-19 infection.^1^ Early studies using aggregate administrative data on national cigarette and tobacco sales documented increases between 12% and 14%.^2–4^ School closures and stay-at-home orders increased children’s potential exposure to secondhand smoke.^5^ These conditions prompted immediate interest in understanding changes in smoking behavior during COVID-19. However, findings from studies that used household-level microdata have not been consistent.^6–10^ The mismatch between national sales data and household microdata studies suggests that which households smoked more during COVID-19 is not yet reliably known. Furthermore, no studies have explored whether smoking increased in households with children, potentially increasing secondhand smoke exposure. This study addressed these gaps by estimating changes in cigarette purchases during the COVID-19 pandemic across different household types, with a focus on parents.

Parents and children faced considerable challenges owing to school and childcare center closures, the switch to distance learning, and remote work.^11,12^ Some who worked in essential sectors had to return to work without reliable child care, whereas others lost their jobs, straining family finances.^13,14^ These factors led to record increases in reports of parental stress and anxiety during COVID-19. Individuals with elevated anxiety-related symptoms are more likely to smoke than those without these symptoms.^15,16^ Relative to the childless, parents’ elevated stress levels during the pandemic may have led to distinct changes in their smoking habits.

These behavioral levers also varied by household race and ethnicity. Black and Hispanic families were more likely to live in remote-only school districts but were also the least likely to have access to the internet or a computer to facilitate remote learning.^17^ The effects of COVID-19 on unemployment also varied by race/ethnicity, with higher unemployment in Black and Hispanic households than in White households,^18^ resulting in disproportionate increases in economic and psychological vulnerability.^19,20^ These facts suggest that changes in smoking behavior during the pandemic may have differed by race and ethnicity and motivate a focus on Black and Hispanic parents, in particular.

The existing literature on smoking during COVID-19 is plagued by mixed results. On the one hand, data on tobacco manufacturers’ monthly filings to the U.S. Department of the Treasury showed that cigarette sales were 14% higher than expected between March 2020 and June 2021.^3^ Using aggregated NielsenIQ Consumer Panel data—the same data used in this study—Lee et al.^21^ (2021) found a 13.2% increase in tobacco spending in April and June 2020 compared with that in the same period in 2019.

On the other hand, analyses using other household-level data sources have differed from national sales data. Studies using representative national surveys such as the Behavioral Risk Factor Surveillance System (BRFSS) and National Health Interview Study primarily focused on smoking prevalence showed no change or small reductions in cigarette smoking prevalence during the pandemic.^7–10^ Fewer studies have explored year-over-year changes in smoking intensity. Using National Health Interview Study data on individuals with chronic lung diseases, Seegulam and colleagues.^22^ (2025) showed a marginally significant increase in the number of daily cigarettes among Black individuals in 2021 and 2023 relative to that in 2019. In contrast, the Consumer Expenditure Diary survey data showed a 15.5% decrease in tobacco expenditures in 2020 compared with that in 2019.^6^ These inconsistencies highlight several gaps in our understanding of smoking behavior during the COVID-19 pandemic.

This study addresses 3 of these gaps. First, changes in smoking behavior during COVID-19 by household characteristics were estimated using the NielsenIQ Homescan Consumer Panel (Consumer Panel). The Consumer Panel data reports household purchases on a weekly basis for a fixed set of panel households, which allowed the same households to be followed over 2019 and 2020. This offers an improvement over existing microdata studies, which do not track the same households over the first months of COVID-19.^6–10^ Second, changes in both smoking prevalence (rates of cigarette purchase) and smoking intensity (number of cigarettes, expenditures) were explored, providing a comprehensive analysis of both extensive and intensive margin changes. Third, pandemic-related changes in smoking behavior among parents were studied. Children’s exposure to secondhand smoke has been linked to growth,^23^ asthma and other acute respiratory symptoms,^24,25^ and academic achievement.^26–28^ Thus, understanding the impact of the pandemic on parental smoking and potential secondhand smoke exposure for children can underscore the need for policies to address parental stress and can inform future debates around school-closure policies in times of public health emergencies.

## METHODS

This study used data from the 2019 and 2020 NielsenIQ Homescan Consumer Panel data, a deidentified survey data set available to researchers for purchase (making the study IRB exempt). The Consumer Panel is a longitudinal data set that tracks household purchases at the transaction level. A stratified, proportionate sampling method is used, and email outreach and incentives are used to recruit and maintain a representative sample. The 1-year retention rate for sample households is about 80%. Households record all items purchased from 67 unique retailer types, including gas and convenience stores, grocery stores, tobacco shops, and online retailers. The transaction data include the quantity and price of each purchased item. Demographic information is gathered once a year from participating households, in the final months of each year. More details about the data sampling frame and accuracy are available in Zhen et al.^29^ and Einav and colleagues.^30^

### Study Sample

The Consumer Panel data for 2019 and 2020 include about 71,000 households. The primary analytical sample included 24,795 households where the average age of household heads was ≤55 years and where at least 1 transaction in each of the pre and postpandemic periods was recorded (demarcated by the U.S. government’s declaration of a national emergency on March 13, 2020). The average household reported transactions in 42 weeks in 2019 and in 35 weeks in 2020, leading to 1,911,675 household-week observations (sample details are presented in Appendix Figure 1, available online).

White, Black, and Hispanic households accounted for 67.5%, 12.2%, and 11.5% of the sample, respectively (Table 1). In analyses by race/ethnicity, the sample was limited to include only the focal group and White families as comparison. Forty-four percent of households included parents; 38.9% had at least 1 child aged between 6 and 17 years (school-aged child), and 5.5% had no school-aged children but had at least 1 child aged ≤5 years (young child). Most households (63.7%) reported at least 1 head with a college degree, and 56.9% included 2 adults.

### Measures

The analysis used 2 primary measures of weekly smoking behavior. First, to measure smoking prevalence, an indicator was created that equaled 1 if a household reported buying any cigarettes in a week. Second, to measure smoking intensity, the total number of cigarettes purchased each week was calculated by counting the number of cigarettes in each transaction and summing across transactions in a week. The number of cigarettes was recorded for households who indicated having bought any cigarettes in the previous 4 weeks (referred to as households that smoked). Total weekly cigarette expenditures conditional on having purchased in the previous 4 weeks were also analyzed (results are presented in the Appendix, available online).

### Statistical Analysis

A series of household fixed-effect regression models were estimated on weekly cigarette purchase outcomes, comparing the same households through 2019 and 2020 across the onset of the pandemic (analyses conducted in 2024 and 2025) (details and models are presented in the Appendix, available online). First, a pre/post model was estimated for the entire sample to document average changes in cigarette purchase behavior at the onset of the pandemic; next, a difference-in-difference model was estimated to evaluate whether parents changed their purchase behavior differently compared with childless households; and finally, difference-in-difference-in-difference (DDD) models were estimated that disaggregated parents by race/ethnicity and by child age. All regressions included fixed effects for households, year (2020 vs 2019), and week-in-year (51 weeks, relative to the first week of the year). Using Version 18 of STATA, ordinary least squares models with household-clustered SEs were estimated, and a *p*=0.05 was used to indicate statistical significance.

## RESULTS

Table 2 shows the means of the weekly cigarette purchase outcomes before (before pandemic) and during (after pandemic) the pandemic for the focal populations. Overall, about 3.6% of households reported buying cigarettes in a week before the onset of the pandemic; 3.9% reported weekly buying during the pandemic. Smoking households reported buying 68 cigarettes in a week before the pandemic and 75 per week during the pandemic. Weekly spending also increased accordingly. The average parent who smoked reported buying an extra 11 cigarettes per week during the pandemic, whereas the average Hispanic parent did not increase cigarette purchases. On average, Black parents who smoked reported buying 15 more cigarettes per week after the onset of the pandemic.

**Figure 1.**
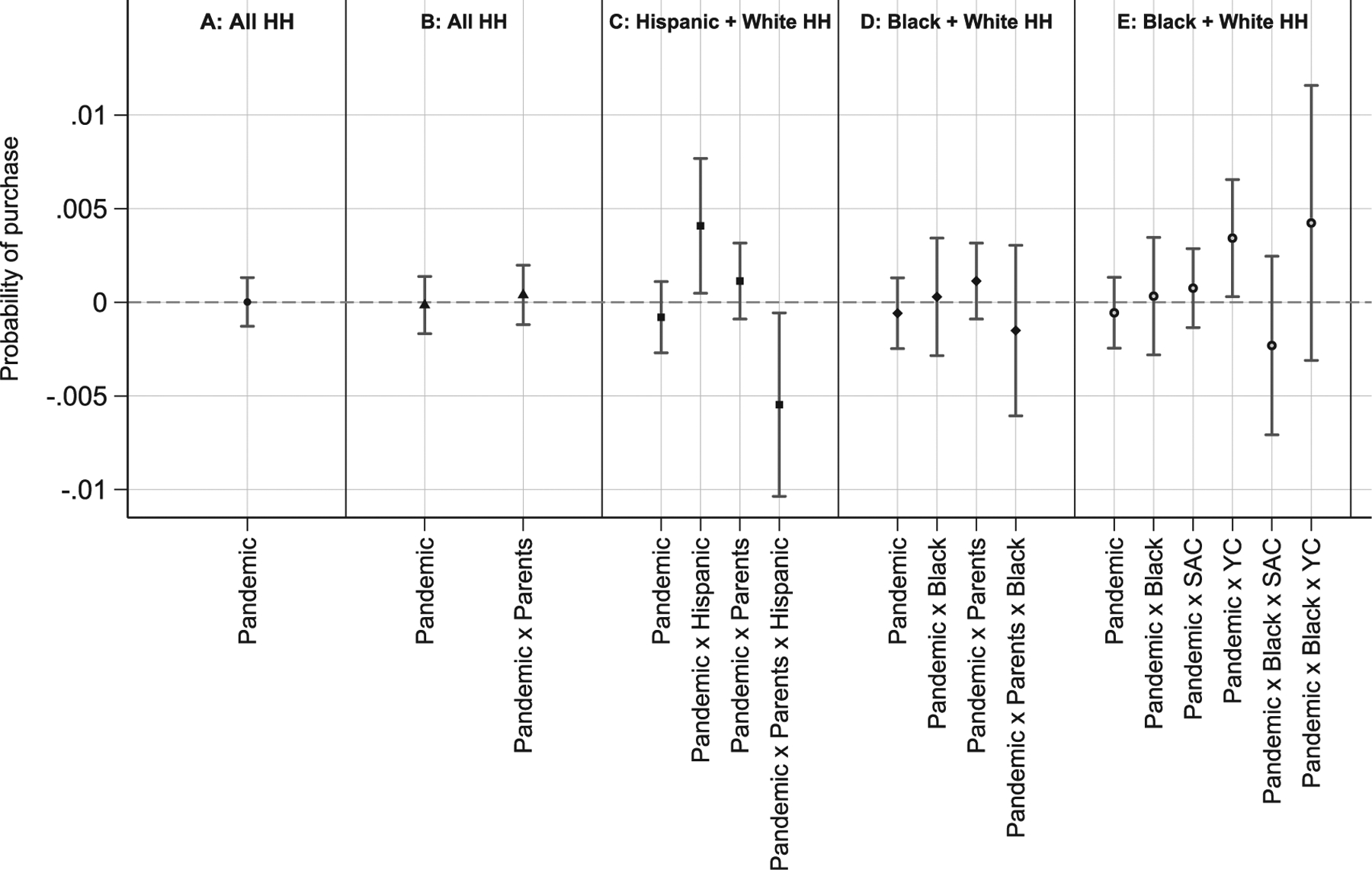
Effect of the pandemic on the likelihood of weekly cigarette purchase, NielsenIQ Homescan Consumer Panel, 2019–2020. *Notes:* Each panel reports results from a separate regression. In Panels A and B, the analytical sample includes households that reported at least 1 transaction in both the pre and postpandemic periods (11th week of 2020) and where the average age of household heads is ≤55 years; analytical samples in Panels C, D, and E are further limited according to the panel header. The outcome variable is a binary indicator of whether a household reported purchasing any cigarettes in a week. Point estimates are presented with 95% CIs, estimated with SEs clustered at the household level. The variables pandemic, parents, Black, Hispanic, SAC, and YC are binary indicators. All models control for time (year, month, week within the year) and household fixed effects as well as the main effect of parent status and race/ethnicity by parent status. Exact point estimates are presented in Appendix Table 1 (available online). SAC, school-aged children; YC, young children. Source: Authors’ analysis of data from the NielsenIQ Homescan Consumer Panel; 2019–2020.

**Figure 2.**
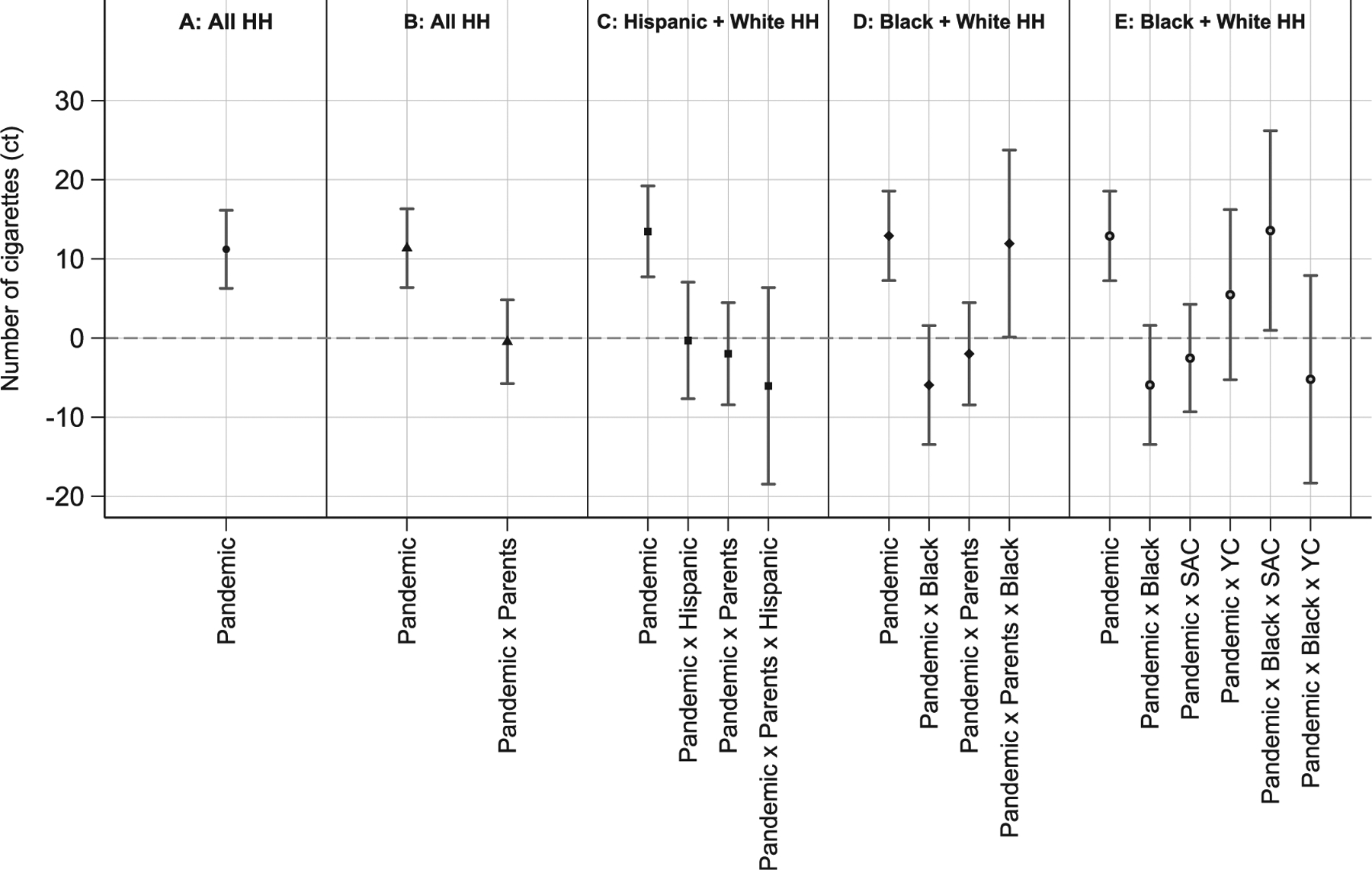
Effects of the pandemic on the number of cigarettes purchased among households that smoked, NielsenIQ Homescan Consumer Panel, 2019–2020. *Notes*: Each panel reports results from a separate regression. In Panels A and B, the analytical sample includes households that reported at least 1 transaction in both the pre and postpandemic period (11th week of 2020), where the average age of household heads is ≤55 years, and where the household reported at least 1 cigarette purchase in the previous 4 weeks; analytical samples in Panels C, D, and E are further limited according to the panel header. The outcome is the weekly number of cigarettes purchased (count). Point estimates are presented with 95% CIs, estimated with SEs clustered at the household level. The variables pandemic, parents, Black, Hispanic, SAC, and YC are binary indicators. All models control for time (year, month, week within the year) and household fixed effects as well as the main effect of parent status and race/ethnicity by parent status. Exact point estimates are presented in Appendix Table 2 (available online). SAC, school-aged children; YC, young children. Source: Authors’ analysis of data from the NielsenIQ Homescan Consumer Panel; 2019–2020.

Figures 1 and 2 illustrate the study findings for the primary outcomes. Each figure plots the coefficient estimates and 95% CIs obtained from estimating separate models for the different focal groups, with estimation samples noted at the top of each panel (exact point estimates in Appendix Tables 1 and 2, available online). Each figure progresses similarly. Panel A shows the estimated change in purchase behavior during the pandemic for the general population. Panel B shows the results of the difference-in-difference model comparing parents with nonparents. The final 3 panels of each figure show the results of the DDD models illustrating racial/ethnic differences, each estimated on the focal group and White households as comparison. For each model, the estimated change in purchase behavior across all households is reported (i.e., the pandemic term), followed by estimates of the additional (marginal) changes in purchase behavior for each focal group (i.e., the pandemic X group interaction terms). Adding the main and relevant interaction coefficients captures the total COVID-19 change in cigarette purchase behavior for each group.

Figure 1 shows no change in the probability of purchasing cigarettes for the full population (Panel A), for parents in general (Panel B), or for Black parents (Panel D). Panel C illustrates that although childless Hispanics households were 0.4 percentage points or 13% more likely to buy cigarettes weekly, the relative change among Hispanic parents was −0.5 percentage points, completely offsetting the increase in childless households. Summing the statistically significant coefficients shows that Hispanic parents were 0.1 percentage points (8%) less likely to purchase cigarettes during the pandemic than before the pandemic period. In the results disaggregated by child age (Panel E), the statistically significant pandemic X young children coefficient indicates that White parents with young children were 0.3 percentage points (26%) more likely to purchase cigarettes during the pandemic.

Figure 2 reports the estimates from models of the conditional number of cigarettes purchased each week. The average household that smoked bought about 11.2 (16%) more cigarettes per week during COVID-19 (Panel A). Panels B and C illustrate that parents who smoked and Hispanic parents who smoked did not change their cigarette consumption differently than the average person who smoked, increasing their weekly purchase amount by about 11–13 cigarettes. However, relative to White parents and childless households, Black parents who smoked purchased an additional 11.9 cigarettes per week during COVID-19 (Panel D). Summing the statistically significant main and marginal effects in this model suggests that Black parents who smoked purchased about 25 more cigarettes per week during the pandemic (67%). The additional increase among Black parents who smoked was concentrated among those with school-aged children in the household (Panel E).

Estimates were robust to the inclusion of additional controls (Appendix Tables 1 and 2, available online) and a Poisson model specification (Appendix Table 3, available online). The results were also generally consistent across a set of alternately specified outcome variables (total weekly cigarette spending, total weekly tobacco spending, share of total weekly expenditures on cigarettes, and cigarettes per adult), although the coefficients on the Black parent group were not statistically significant (Appendix Figure 2, available online, and Appendix Tables 4 and 5, available online). The relative likelihood of buying nicotine replacement therapy products increased among White parents who smoked and decreased among Black parents who smoked (Appendix Tables 5, available online).

Analyses were conducted to explore potential mechanisms. First, the average price per pack dropped by about 15 cents during COVID-19, with no differences across groups, ruling out the possibility that prices drove changing purchase behavior (Appendix Table 5, available online). To rule out stockpiling—fewer shopping trips with more cigarettes per trip—following Lee et al.^21^ (2021), the household-level data were aggregated for the April–December 2019 and 2020 periods (Appendix Table 6, available online). *t*-tests comparing the 2019 with 2020 periods showed a statistically significant but small increase in the probability of buying any cigarettes during the pandemic (9.4% of households in 2019 and 10.5% in 2020), increases in the total number of cigarettes purchased among households that smoked (1,142 vs 1,299 cigarettes [14%], overall), and a statistically insignificant but relatively large increase in the total number of cigarettes among Black parents who smoked (612 vs 789 cigarettes [29%]).

To test whether the observed racial/ethnic differences during the pandemic were driven by existing disparities in smoking rates by SES,^31^ DDD models were estimated replacing the racial/ethnic indicators with an indicator for low education (high school or less) (Appendix Table 7, available online). Although childless households with low education reduced their purchase probability by about 0.6 percentage points, parents with low education who had young children were 86% more likely to purchase cigarettes in a week during COVID-19. Otherwise, compared with higher-education households, households with lower education did not exhibit differential changes in COVID-19 smoking prevalence or intensity. Finally, older households with heads aged between 55 and 75 years exhibited behavior similar to that of younger households, with no change in smoking prevalence and an increase in intensity among households that smoked (Appendix Table 8, available online).

## DISCUSSION

Aggregate cigarette sales increased between 12% and 14% during the 2020 pandemic.^2–4^ This study’s results suggest that the increase was not driven by changing prevalence: the fixed-effects models show that the probability of buying cigarettes did not increase among the full sample and that the probability decreased among Hispanic parents. Parents of young children were an exception: White parents of young children and low-education parents of young children were 26% and 86% more likely to purchase cigarettes in a week, respectively. Childless Hispanic households also increased their purchase probability by about 13%.

Rather, this study documents that the increase in aggregate sales was driven by households that smoked, with the average household buying an extra half a pack per week. Disaggregated estimates by parental status and by race/ethnicity show the largest relative increases in the number of cigarettes purchased among Black households. Whereas White parents who smoked bought about an extra half pack per week, Black parents nearly doubled their weekly purchases from about 1 pack per week to 2 packs per week. Although the prevalence of smoking decreased from 2011 to 2020 for both Black and White adults,^10^ the relative decline was larger among White adults (20.6%–13.3%) than among Black adults (19.4%–14.4%).^32^ The results of this study suggest that the unique economic, social, and school-related stressors that Black households with children faced during the pandemic may have further worsened racial disparities in smoking.

Black parents with school-aged children exhibited the largest growth in smoking intensity, and prevalence increased among White and low-education parents of young children. These findings point to the extreme increases in stress that parents experienced during the pandemic owing to school closures, stay-at-home orders, economic uncertainty, and fear of the virus itself.^33^ Many studies indicate that alcohol consumption among parents increased during COVID-19, with some parents using alcohol to cope with pandemic-related stress, loneliness, and depression.^34^ This study shows that cigarette consumption also increased for some parents, suggesting the need for policy that addresses parental stress in times of public health emergencies.

The results in this study align with some national survey data. For example, BRFSS data showed a reduction in smoking prevalence among Hispanic households,^7^ aligning with the estimated reduction among Hispanic parents. Data from BRFSS also showed reduced quit attempts among Black households.^35^ However, the results differ from those of studies of smoking intensity over COVID-19.^6,9^ The household fixed-effect models used in this study help to explain these differences because within-household estimates are less susceptible to pandemic-related sample changes. Most importantly, the present results align with aggregate sales data and illustrate which households drove the 14% increase in aggregate cigarette spending observed in the early months of COVID-19.^3–5^

### Limitations

The Consumer Panel data have limitations. First, women and higher-educated households are over-represented in the Consumer Panel.^29^ Second, transactions are not always accurately reported, with some missing and others misreported.^30^ Third, fewer households reported transactions in 2020 than in 2019, suggesting that some may have left the panel in response to the pandemic. These factors limit the external validity of the results. However, given that the estimates align with data from trusted federal sources, misreporting does not appear to systematically differ by smoking status or over time.^2–4^ Finally, during COVID-19, the number of children living in multigenerational households grew by about 10% among Black and Hispanic families, while remaining nearly flat among White families.^36^ Because the Consumer Panel measure of household composition—collected in late 2019—does not capture these changes, the results do not necessarily imply that Black parents smoked more: increased cigarette purchases may simply reflect new adults living in households. However, it is more certain that children living in these households had increased exposure to smoking.

## CONCLUSIONS

Using the Nielsen IQ Consumer Panel data, a data source that aligns with national cigarette sales data, this study documented demographic patterns in cigarette purchases during the first months of the COVID-19 pandemic. There was limited evidence that nonsmoking households took up smoking during COVID-19. However, relative to before the pandemic, the average smoking household bought about an extra half a pack of cigarettes per week. Notably, Black parents of school-aged children who smoked nearly doubled their cigarette purchases, buying an extra pack per week. The additional cigarettes in these households create the potential for significant increases in secondhand smoke exposure among Black children. Given the short- and long-term health and cognitive impacts of secondhand smoke exposure, this represents a significant public health concern.

## Supplementary Material

1

Supplemental materials associated with this article can be found in the online version at https://doi.org/10.1016/j.amepre.2025.107980.

## Figures and Tables

**Table 1.** Descriptive Statistics of the Sample Households, NielsenIQ Homescan Consumer Panel Households, 2019–2020

Characteristics	% of the sample
Representative race of household	
White	67.5
Black	12.2
Other	8.8
Hispanic	11.5
Highest education	
High school or less	12.0
Some college	24.3
College or more	63.7
Age of heads, years	
≤35	13.3
35≤45	37.0
45≤50	23.7
50≤55	26.0
Number of adults	
1 person	20.6
2 people	56.9
3 people	15.2
4 people or more	7.4
Parental status	
Childless	55.6
Any school-aged children	38.9
Only young children	5.5
*n* (household-week)	1,911,675
*n* (household)	24,795

Source: Authors’ analysis of data from the NielsenIQ Homescan Consumer Panel; 2019–2020

*Note:* The analytical sample includes households that reported at least 1 transaction in both the pre and postpandemic period (11th week of 2020) and where the average age of household heads is ≤55 years. Race of the household is the household’s reported representative race. If a household’s reported race/ethnicity differed between 2019 and 2020, the 2020 race/ethnicity was used. The White, Black, and other categories are exclusive of Hispanics. For households with 2 heads, highest education is the highest level of education between the 2 partners, and the age of the heads is their average age. Childless households do not have any children aged <18 years living in the household. Parents of school-aged children have at least 1 school-aged child (≥6 years). Parents of young children have only younger children living at home (≤5 years).

**Table 2.** Average Weekly Cigarette Purchase Outcomes, NielsenIQ Homescan Consumer Panel Households, 2019–2020

Weekly cigarette purchase outcome	Prepandemic mean	Postpandemic mean
Overall		
Buying (binary)	0.036	0.039
Number (count; buyer=1)	68.08	74.94
Spending ($; buyer=1)	20.75	23.66
Parents		
Buying (binary)	0.021	0.026
Number (count; buyer=1)	58.35	69.01
Spending ($; buyer=1)	17.59	21.82
Black parents		
Buying (binary)	0.022	0.020
Number (count; buyer=1)	36.93	52.13
Spending ($; buyer=1)	12.64	17.06
Hispanic parents		
Buying (binary)	0.0183	0.0177
Number (count; buyer=1)	38.00	34.92
Spending ($; buyer=1)	12.75	12.70

Source: Authors’ analysis of data from the NielsenIQ Homescan Consumer Panel; 2019–2020.

*Notes:* The analytical sample includes households that reported at least 1 transaction in both the pre and postpandemic period (11th week of 2020) and where the average age of household heads is ≤55 years. The prepandemic period is defined as spanning from the 4th week of 2019 to the 10th week of 2020, whereas the postpandemic period covers the 11th to the 52nd week of 2020. The number and spending variables are conditional on having made any cigarette purchases in the previous 4 weeks.
