# Supplementary material for: Parents’ Cigarette Purchases During COVID-19: Evidence From NielsenIQ Consumer Panel Data
Source: Am J Prev Med. Author manuscript; Available in PMC 2026 May 6. (PMC13147754; doi:10.1016/j.amepre.2025.107980)
Supplement: 1 [file NIHMS2169659-supplement-1.docx]

**American Journal of Preventive Medicine**

**Online Appendix to**

**Parents’ Cigarette Purchase Behavior during COVID-19: Evidence from NielsenIQ Consumer Panel Data**

Lauren E. Jones, PhD, Nahae Kang, MS, and Tansel Yilmazer, PhD

**Sample Details and Variable Construction**

Appendix Figure 1 illustrates the number of survey households that were dropped with each sample restriction imposed.

Appendix Figure 1. Details of sample construction


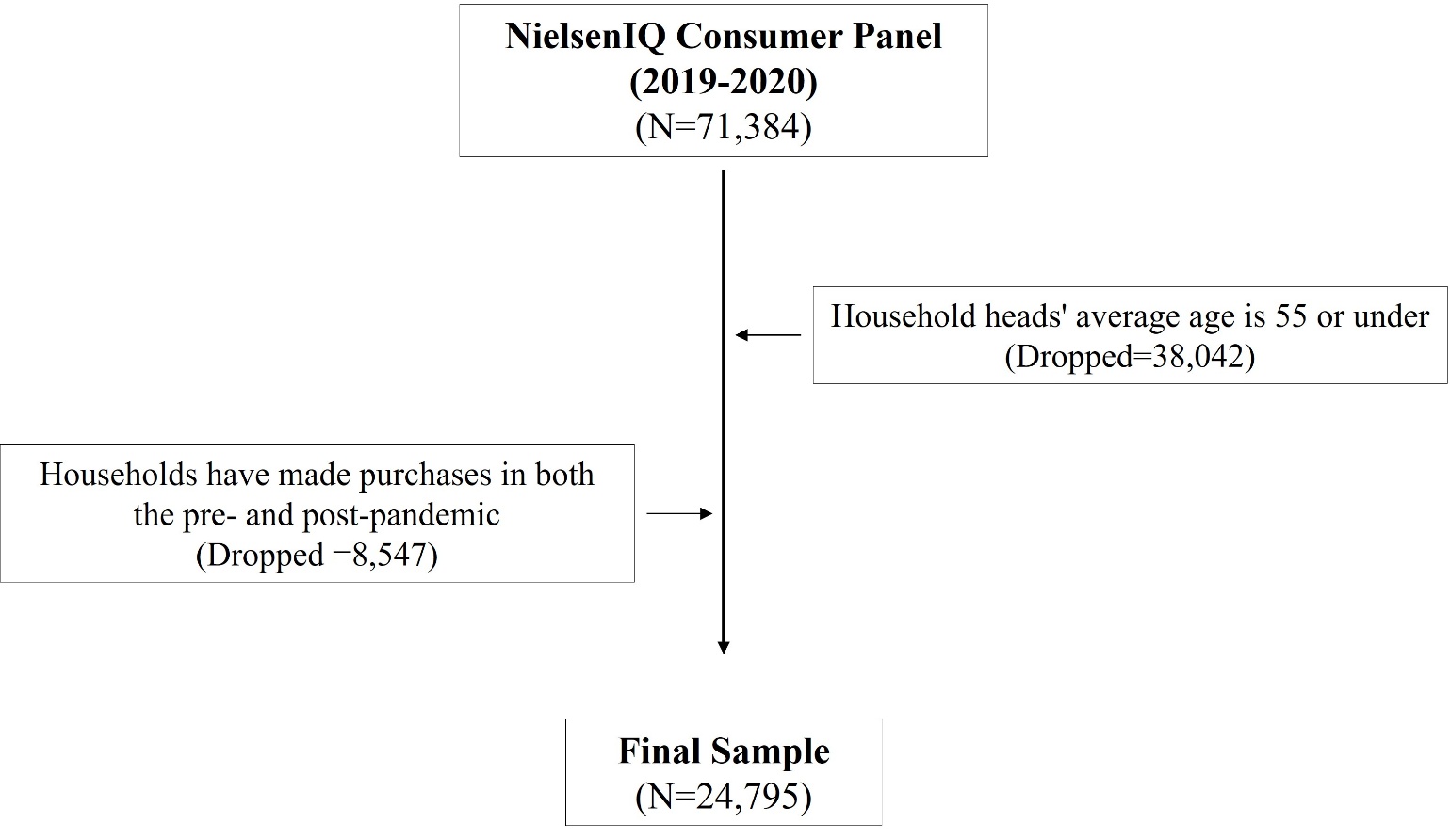


To identify race and ethnicity, the question that asked household heads about the household’s representative race was used. Households were classified according to the categories: Non-Hispanic White, Non-Hispanic Black, Non-Hispanic Other, and Hispanic. Parent households were defined as households that included a child under age 18 who was reported as a son or daughter of a household head. The highest education variable refers to the highest level of education attained by any household head. The age of household heads is defined as the average age when a household has two heads, and as the head’s age in single-head households. The number of adults includes all household heads and household members aged 18 or older.

**Difference-in-difference-in-difference approach**

To estimate the impact of the pandemic on cigarette purchase behavior, a stepwise analysis was conducted wherein interaction terms were sequentially added to a pre/post regression model, building up to a difference-in-difference-in-difference (DDD) model.

The first model for estimating the effect of the pandemic on overall cigarette purchases can be expressed as follows:

| ${SO}_{iyw}=\beta_{0}+\beta_{1}{Pandemic}_{yw}+\delta_{y}+\mu_{w}+\sigma_{i}+\varepsilon_{iyw}$ | (1) |
| --- | --- |

where ${SO}_{iyw}$ represents smoking outcome for household *i*, in week *w* (i.e., 2 - 52 weeks), in year *y* (i.e. 2020 versus 2019). ${Pandemic}_{yw}$ is a binary variable that equals 1 beginning the week of the declaration of a national emergency (week 11 of 2020), and thereafter. Fixed effect controls for year ($\delta_{y}$), week-in-year ($\mu_{w}$), and households ($\sigma_{i}$) were included. Finally, $\varepsilon_{iyw}$ is an error term. The coefficient $\beta_{1}$ indicates the average change in smoking outcomes after the pandemic in the full population.

The second model examined the COVID-19 change in cigarette purchase behavior among parents using a difference-in-difference (DD) approach. It is specified as follows:

| ${SO}_{iyw}=\beta_{0}+\beta_{1}{Pandemic}_{yw}+\beta_{2}\left( {Pandemic}_{yw}\times{Parents}_{iy} \right)+\beta_{3}{Parents}_{iy}$  $+\delta_{y}+\mu_{w}+\sigma_{i}+\varepsilon_{iyw}$ | (2) |
| --- | --- |

Here, ${Parents}_{iy}$ is a binary indicator for households with children aged 17 and under. It is interacted with the ${Pandemic}_{yw}$ variable. Other variables are equivalent to Eq. (1). The coefficient $\beta_{1}$ indicates the average change in smoking outcomes after the pandemic across the full population, and $\beta_{2}$ indicates any additional (marginal) increase among parents relative to non-parents. The coefficient $\beta_{3}$ captures pre-pandemic differences in purchase outcomes between parents and non-parents.

The coefficient $\beta_{1}$ alone captures the COVID-19 change in purchase behavior among non-parents. The total COVID-19 change in purchase behavior among parents can be evaluated by summing coefficients $\beta_{1}$ and $\beta_{2}.$ Note that if the estimates of $\beta_{1}$ or $\beta_{2}$ are statistically insignificant, they are assumed to be equal to 0.

The third model was used to evaluate racial/ethnic differences in cigarette purchase behavior. Estimated on the focal racial/ethnic group (Black or Hispanic) and white households for comparison, the DDD model is specified as follows:

| ${SO}_{iyw}=\beta_{0}+\beta_{1}{Pandemic}_{yw}+\beta_{2}\left( {Pandemic}_{yw}\times{Parents}_{iy} \right)+\beta_{3}\left( {Pandemic}_{yw}\times{Race}_{i} \right)+\beta_{4}\left( {Pandemic}_{yw}\times{Parents}_{iy}\times{Race}_{i} \right)+\beta_{5}\left( {Parents}_{iy}\times{Race}_{i} \right)+\beta_{6}{Parents}_{iy} +\delta_{y}+\mu_{w}+\sigma_{i}+\varepsilon_{iyw}$ | (3) |
| --- | --- |

where ${Race}_{i}$ is an indicator for households that are Black or Hispanic (according to self-report in late 2019), relative to white households. The racial/ethnic indicator is interacted with the ${Pandemic}_{yw}$ and ${Parents}_{iy}$ terms from (2). Other variables are equivalent to Eq. (1). Note that the model does not include a main effect control for ${Race}_{i}$, since it does not vary within households across years, and is therefore absorbed by the household fixed effect.

The coefficient $\beta_{1}$ indicates the average change in smoking outcomes after the pandemic across the full population, $\beta_{2}$ indicates any additional (marginal) increase among parents relative to non-parents, and $\beta_{3}$ indicates any additional change among the focal racial/ethnic group relative to white households. Finally, coefficient $\beta_{4}$ on the triple interaction reflects the marginal post-pandemic change after the pandemic among parents in the focal racial/ethnic group, relative to the changes among childless households, and white parents. Coefficients $\beta_{5}$ and $\beta_{6}$ reflect differences in average smoking outcomes across groups in the pre-pandemic era.

In this model, the coefficient $\beta_{1}$ alone captures the COVID-19 change in purchase outcomes for white non-parents. The COVID-19 change in purchase behavior among white parents can be evaluated by summing coefficients $\beta_{1}$ and $\beta_{2}.$ The change among non-parents in the focal racial/ethnic group is captured by summing coefficients $\beta_{1}$ and $\beta_{3}.$ The total change among parents in the focal racial/ethnic group is evaluated by summing coefficients $\beta_{1}$, $\beta_{2}$, $\beta_{3},$ and $\beta_{4}$. Note that if any of the estimates of $\beta_{1}$ through $\beta_{4}$ are statistically insignificant, they are assumed to be equal to 0.

Finally, to examine the effect of the pandemic on cigarette purchases by child age, the following model was used:

| ${SO}_{iyw}=\beta_{0}+\beta_{1}{Pandemic}_{yw}+\beta_{2}\left( {Pandemic}_{yw}\times{Race}_{i} \right)+\beta_{3}\left( {Pandemic}_{yw}\times{YC}_{iy} \right)+\beta_{4}\left( {Pandemic}_{yw}\times{SAC}_{iy} \right)+\beta_{5}\left( {Pandemic}_{yw}\times{YC}_{iy}\times{Race}_{i} \right)+\beta_{6}\left( {Pandemic}_{yw}\times{SAC}_{iy}\times{Race}_{i} \right)+\beta_{7}\left( {SAC}_{iy}\times{Race}_{i} \right)+\beta_{8}\left( {YC}_{iy}\times{Race}_{i} \right)+\beta_{9}{SAC}_{iy}+\beta_{10}{YC}_{iy}$  $+\delta_{y}+\mu_{w}+\sigma_{i}+\varepsilon_{iyw}$ | (4) |
| --- | --- |

where ${SAC}_{iy}$ is a binary indicator for households with any children aged 6 to 17 and ${YC}_{iy}$ indicates households that had only children under 6. The sample is restricted to white (reference) and Black or Hispanic households.

The coefficient $\beta_{1}$ indicates the average change in smoking outcomes after the pandemic across the full population. The coefficient $\beta_{2}$ indicates any additional (marginal) increase among non-parents in the focal racial/ethnic group relative to white households. Coefficients $\beta_{3}$ and $\beta_{4}$ indicate additional COVID-19 changes in purchase outcomes among white parents of young and school aged children, respectively. Coefficients $\beta_{5}$ and $\beta_{6}$ capture the marginal COVID-19 changes in purchase outcomes for parents in the focal racial/ethnic group of young and school-aged children, respectively. Coefficients $\beta_{7}$ through $\beta_{10}$ reflect differences in average smoking outcomes across groups in the pre-pandemic era.

In this model, the coefficient $\beta_{1}$ alone captures the COVID-19 change in purchase outcomes for white non-parents; the sum of $\beta_{1}$ and $\beta_{2}$ captures the change among childless households in the racial/ethnic focal group. The COVID-19 change in purchase behavior among white parents of young children can be evaluated by summing coefficients $\beta_{1}$ and $\beta_{3}$; and the change among white parents of school aged children is the sum of $\beta_{1}$ and $\beta_{4}$. Finally, the total change among parents with young children in the focal racial/ethnic group is evaluated by summing coefficients $\beta_{1}$, $\beta_{2}$, $\beta_{3}$, and $\beta_{5}$; and the total change for parents of school-aged children in the racial/ethnic group is the sum of $\beta_{1}$, $\beta_{2}$, $\beta_{4}$, and $\beta_{6}$. Note that if any of the estimates of $\beta_{1}$ through $\beta_{6}$ are statistically insignificant, they are assumed to be equal to 0 in evaluating the group-specific changes.

Appendix Table 1. Estimation Results, Probability of Buying any Cigarettes in a Week

|  | Baseline | State SxY FE |  | Baseline | State SxY FE |  | Baseline | State SxY FE |  | Baseline | State SxY FE |  | Baseline | State SxY FE |  | Baseline | State SxY FE |
| --- | --- | --- | --- | --- | --- | --- | --- | --- | --- | --- | --- | --- | --- | --- | --- | --- | --- |
|  | (1) | (2) |  | (3) | (4) |  | (5) | (6) |  | (7) | (8) |  | (9) | (10) |  | (11) | (12) |
| Pandemic | 0.00002 | 0.00002 |  | -0.00015 | -0.00014 |  | -0.00080 | -0.00074 |  | -0.00059 | -0.00062 |  | -0.00056 | -0.00059 |  | -0.00077 | -0.00072 |
| (pre-pandemic : 0.03) | (0.00066) | (0.00066) |  | (0.00078) | (0.00078) |  | (0.00097) | (0.00097) |  | (0.00096) | (0.00097) |  | (0.00097) | (0.00097) |  | (0.00097) | (0.00097) |
|  |  |  |  |  |  |  |  |  |  |  |  |  |  |  |  |  |  |
| Pandemic x Hispanic |  |  |  |  |  |  | 0.00408* | 0.00401* |  |  |  |  |  |  |  | 0.00409* | 0.00403* |
| (pre-pandemic, Hispanic : 0.03) |  |  |  |  |  |  | (0.00184) | (0.00182) |  |  |  |  |  |  |  | (0.00184) | (0.00182) |
|  |  |  |  |  |  |  |  |  |  |  |  |  |  |  |  |  |  |
| Pandemic X Black |  |  |  |  |  |  |  |  |  | 0.00029 | 0.00087 |  | 0.00033 | 0.00091 |  |  |  |
| (pre-pandemic, Black : 0.02) |  |  |  |  |  |  |  |  |  | (0.00160) | (0.00162) |  | (0.00160) | (0.00162) |  |  |  |
|  |  |  |  |  |  |  |  |  |  |  |  |  |  |  |  |  |  |
| Pandemic x Parents |  |  |  | 0.00039 | 0.00037 |  | 0.00113 | 0.00101 |  | 0.00113 | 0.00100 |  |  |  |  |  |  |
| (pre-pandemic, parents : 0.02) |  |  |  | (0.00081) | (0.00082) |  | (0.00103) | (0.00105) |  | (0.00103) | (0.00105) |  |  |  |  |  |  |
|  |  |  |  |  |  |  |  |  |  |  |  |  |  |  |  |  |  |
| Pandemic x Parents x Hispanic |  |  |  |  |  |  | -0.00546* | -0.00527* |  |  |  |  |  |  |  |  |  |
| (pre-pandemic, parents, Hispanic : 0.02) |  |  |  |  |  |  | (0.00250) | (0.00250) |  |  |  |  |  |  |  |  |  |
|  |  |  |  |  |  |  |  |  |  |  |  |  |  |  |  |  |  |
| Pandemic x Parents x Black |  |  |  |  |  |  |  |  |  | -0.00151 | -0.00143 |  |  |  |  |  |  |
| (pre-pandemic, parents, Black : 0.02) |  |  |  |  |  |  |  |  |  | (0.00232) | (0.00233) |  |  |  |  |  |  |
|  |  |  |  |  |  |  |  |  |  |  |  |  |  |  |  |  |  |
| Pandemic x SAC |  |  |  |  |  |  |  |  |  |  |  |  | 0.00076 | 0.00068 |  | 0.00076 | 0.00070 |
| (pre-pandemic, parents, SAC : 0.02) |  |  |  |  |  |  |  |  |  |  |  |  | (0.00108) | (0.00109) |  | (0.00108) | (0.00109) |
|  |  |  |  |  |  |  |  |  |  |  |  |  |  |  |  |  |  |
| Pandemic x YC |  |  |  |  |  |  |  |  |  |  |  |  | 0.00343* | 0.00287 |  | 0.00343* | 0.00285 |
| (pre-pandemic, parents, YC : 0.01) |  |  |  |  |  |  |  |  |  |  |  |  | (0.00160) | (0.00154) |  | (0.00160) | (0.00154) |
|  |  |  |  |  |  |  |  |  |  |  |  |  |  |  |  |  |  |
| Pandemic x Black x SAC |  |  |  |  |  |  |  |  |  |  |  |  | -0.00231 | -0.00226 |  |  |  |
| (pre-pandemic, Black parents, SAC : 0.02) |  |  |  |  |  |  |  |  |  |  |  |  | (0.00243) | (0.00244) |  |  |  |
|  |  |  |  |  |  |  |  |  |  |  |  |  |  |  |  |  |  |
| Pandemic x Black x YC |  |  |  |  |  |  |  |  |  |  |  |  | 0.00424 | 0.00440 |  |  |  |
| (pre-pandemic, Black parents, YC : 0.02) |  |  |  |  |  |  |  |  |  |  |  |  | (0.00375) | (0.00373) |  |  |  |
|  |  |  |  |  |  |  |  |  |  |  |  |  |  |  |  |  |  |
| Pandemic x Hispanic x SAC |  |  |  |  |  |  |  |  |  |  |  |  |  |  |  | -0.00449 | -0.00438 |
| (pre-pandemic, Hispanic parents, SAC : 0.02) |  |  |  |  |  |  |  |  |  |  |  |  |  |  |  | (0.00251) | (0.00250) |
|  |  |  |  |  |  |  |  |  |  |  |  |  |  |  |  |  |  |
| Pandemic x Hispanic x YC |  |  |  |  |  |  |  |  |  |  |  |  |  |  |  | -0.01242 | -0.01184 |
| (pre-pandemic, Hispanic parents, YC : 0.02) |  |  |  |  |  |  |  |  |  |  |  |  |  |  |  | (0.00748) | (0.00747) |
|  |  |  |  |  |  |  |  |  |  |  |  |  |  |  |  |  |  |
| Observations | 1,911,675 | 1,911,675 |  | 1,911,675 | 1,911,675 |  | 1,509,725 | 1,509,725 |  | 1,523,803 | 1,523,803 |  | 1,523,803 | 1,523,803 |  | 1,509,725 | 1,509,725 |
| Number of households | 24,795 | 24,795 |  | 24,795 | 24,795 |  | 19,568 | 19,568 |  | 19,696 | 19,696 |  | 19,696 | 19,696 |  | 19,568 | 19,568 |
| **SOURCE** Authors' analysis of data from the NielsenIQ Homescan Consumer Panel, 2019-2020. **NOTES** *** p<0.001, ** p<0.01, * p<0.05. Household-clustered standard errors are in parentheses. Analytical sample includes households that reported at least one transaction in both the pre- and post-pandemic period (11th week of 2020), and where the average age of household heads is 55 or younger. The outcome variable is a binary indicator of whether a household reported purchasing any cigarettes in a week. Columns (1), (3), (5), (7), (9) and (11) represent the baseline model, which controls time (year, week in year), and household fixed effects. Columns (2), (4), (6), (8), (10) and (12) include household characteristics, state, state-year fixed effect in addition to the baseline model specifications. All models also include controls for the main effect of parent status and race/ethnicity-by-parent status. The baseline model results align with Figure 1. SAC stands for school-aged children (aged 6 to 17) and YC is young children (under 6). Pre-pandemic means of the dependent variables are provided below DD and DDD coefficients. | | | | | | | | | | | | | | | | | |

Appendix Table 2. Estimation Results, Conditional Number of Cigarettes (count)

|  | Baseline | State SxY FE |  | Baseline | State SxY FE |  | Baseline | State SxY FE |  | Baseline | State SxY FE |  | Baseline | State SxY FE |  | Baseline | State SxY FE |
| --- | --- | --- | --- | --- | --- | --- | --- | --- | --- | --- | --- | --- | --- | --- | --- | --- | --- |
|  | (1) | (2) |  | (3) | (4) |  | (5) | (6) |  | (7) | (8) |  | (9) | (10) |  | (11) | (12) |
| Pandemic | 11.212*** | 11.149*** |  | 11.350*** | 11.509*** |  | 13.472*** | 13.982*** |  | 12.912*** | 13.279*** |  | 12.893*** | 13.248*** |  | 13.443*** | 13.971*** |
| (pre-pandemic : 63.25) | (2.514) | (2.494) |  | (2.534) | (2.540) |  | (2.932) | (2.934) |  | (2.885) | (2.880) |  | (2.887) | (2.880) |  | (2.936) | (2.932) |
|  |  |  |  |  |  |  |  |  |  |  |  |  |  |  |  |  |  |
| Pandemic x Hispanic |  |  |  |  |  |  | -0.311 | -1.041 |  |  |  |  |  |  |  | -0.527 | -1.064 |
| (pre-pandemic, Hispanic : 50.31) |  |  |  |  |  |  | (3.755) | (3.948) |  |  |  |  |  |  |  | (3.767) | (3.950) |
|  |  |  |  |  |  |  |  |  |  |  |  |  |  |  |  |  |  |
| Pandemic x Black |  |  |  |  |  |  |  |  |  | -5.940 | -4.044 |  | -5.934 | -4.066 |  |  |  |
| (pre-pandemic, Black : 51.61) |  |  |  |  |  |  |  |  |  | (3.833) | (3.599) |  | (3.833) | (3.603) |  |  |  |
|  |  |  |  |  |  |  |  |  |  |  |  |  |  |  |  |  |  |
| Pandemic x Parents |  |  |  | -0.469 | -1.238 |  | -1.986 | -3.940 |  | -1.993 | -4.025 |  |  |  |  |  |  |
| (pre-pandemic, parents : 53.42) |  |  |  | (2.696) | (2.498) |  | (3.292) | (2.935) |  | (3.292) | (2.939) |  |  |  |  |  |  |
|  |  |  |  |  |  |  |  |  |  |  |  |  |  |  |  |  |  |
| Pandemic x Parents x Hispanic |  |  |  |  |  |  | -6.040 | -0.901 |  |  |  |  |  |  |  |  |  |
| (pre-pandemic, parents, Hispanic : 38) |  |  |  |  |  |  | (6.332) | (5.464) |  |  |  |  |  |  |  |  |  |
|  |  |  |  |  |  |  |  |  |  |  |  |  |  |  |  |  |  |
| Pandemic x Parents x Black |  |  |  |  |  |  |  |  |  | 11.931* | 14.604* |  |  |  |  |  |  |
| (pre-pandemic, parents, Black : 36.93) |  |  |  |  |  |  |  |  |  | (6.029) | (6.774) |  |  |  |  |  |  |
|  |  |  |  |  |  |  |  |  |  |  |  |  |  |  |  |  |  |
| Pandemic x SAC |  |  |  |  |  |  |  |  |  |  |  |  | -2.538 | -4.574 |  | -2.530 | -4.516 |
| (pre-pandemic, parents, SAC : 57.62) |  |  |  |  |  |  |  |  |  |  |  |  | (3.465) | (3.060) |  | (3.464) | (3.051) |
|  |  |  |  |  |  |  |  |  |  |  |  |  |  |  |  |  |  |
| Pandemic x YC |  |  |  |  |  |  |  |  |  |  |  |  | 5.474 | 3.877 |  | 5.472 | 4.235 |
| (pre-pandemic, parents, YC : 42.73) |  |  |  |  |  |  |  |  |  |  |  |  | (5.480) | (6.174) |  | (5.477) | (6.189) |
|  |  |  |  |  |  |  |  |  |  |  |  |  |  |  |  |  |  |
| Pandemic x Black x SAC |  |  |  |  |  |  |  |  |  |  |  |  | 13.583* | 16.908* |  |  |  |
| (pre-pandemic, Black parents, SAC : 36.98) |  |  |  |  |  |  |  |  |  |  |  |  | (6.431) | (7.335) |  |  |  |
|  |  |  |  |  |  |  |  |  |  |  |  |  |  |  |  |  |  |
| Pandemic x Black x YC |  |  |  |  |  |  |  |  |  |  |  |  | -5.212 | -8.419 |  |  |  |
| (pre-pandemic, Black parents, YC : 36.45) |  |  |  |  |  |  |  |  |  |  |  |  | (6.688) | (8.503) |  |  |  |
|  |  |  |  |  |  |  |  |  |  |  |  |  |  |  |  |  |  |
| Pandemic x Hispanic x SAC |  |  |  |  |  |  |  |  |  |  |  |  |  |  |  | -3.521 | 0.081 |
| (pre-pandemic, Hispanic parents, SAC : 34.9) |  |  |  |  |  |  |  |  |  |  |  |  |  |  |  | (5.836) | (5.642) |
|  |  |  |  |  |  |  |  |  |  |  |  |  |  |  |  |  |  |
| Pandemic x Hispanic x YC |  |  |  |  |  |  |  |  |  |  |  |  |  |  |  | -14.434 | -12.157 |
| (pre-pandemic, Hispanic parents, YC : 59.69) |  |  |  |  |  |  |  |  |  |  |  |  |  |  |  | (10.980) | (10.876) |
|  |  |  |  |  |  |  |  |  |  |  |  |  |  |  |  |  |  |
| Observations | 101,284 | 101,284 |  | 101,284 | 101,284 |  | 85,946 | 85,946 |  | 85,862 | 85,862 |  | 85,862 | 85,862 |  | 85,946 | 85,946 |
| Number of households | 3,317 | 3,317 |  | 3,317 | 3,317 |  | 2,765 | 2,765 |  | 2,742 | 2,742 |  | 2,742 | 2,742 |  | 2,765 | 2,765 |
| **SOURCE** Authors' analysis of data from the NielsenIQ Homescan Consumer Panel, 2019-2020. **NOTES** Household-clustered standard errors are in parentheses. Analytical sample includes households that reported at least one transaction in both the pre- and post-pandemic period (11th week of 2020), where the average age of household heads is 55 or younger, and with a record of purchasing cigarettes in the past four weeks. The outcome is the weekly volume of cigarette purchases (count). Columns (1), (3), (5), (7), (9), and (11) represent the baseline model, which controls time (year, week in year), and household fixed effects. Columns (2), (4), (6), (8), (10), and (12) include household characteristics, state, state-year fixed effect in addition to the baseline model specifications. All models also include controls for the main effect of parent status and race/ethnicity-by-parent status. The baseline model results align with Figure 2. SAC stands for school-aged children (aged 6 to 17) and YC is young children (under 6). Pre-pandemic means of the dependent variables are provided below DD and DDD coefficients. | | | | | | | | | | | | | | | | | |

Appendix Table 3. Estimation Results, Conditional Number of Cigarettes, Poisson Model

|  |  |  |
| --- | --- | --- |
|  | (1) | (2) |
|  |  |  |
| Pandemic | 1.196*** | 1.197*** |
|  | (0.0479) | (0.0480) |
|  |  |  |
| Pandemic x Black | 0.923 | 0.923 |
|  | (0.0646) | (0.0646) |
|  |  |  |
| Pandemic x Parents | 0.979 |  |
|  | (0.0451) |  |
|  |  |  |
| Pandemic x Parents x Black | 1.272* |  |
|  | (0.145) |  |
|  |  |  |
| Pandemic x SAC |  | 0.969 |
|  |  | (0.0462) |
|  |  |  |
| Pandemic x YC |  | 1.260* |
|  |  | (0.136) |
|  |  |  |
| Pandemic x Black x SAC |  | 1.307* |
|  |  | (0.158) |
|  |  |  |
| Pandemic x Black x YC |  | 0.781 |
|  |  | (0.104) |
|  |  |  |
|  |  |  |
| Observations | 85,810 | 85,810 |
| Number of households | 2,720 | 2,720 |
|  |  |  |
| **SOURCE** Authors' analysis of data from the NielsenIQ Homescan Consumer Panel, 2019-2020.  **NOTES** *** p<0.001, ** p<0.01, * p<0.05. Robust standard errors are in parentheses. Coefficients represent incident rate ratios estimated using a Poisson model. Analytical sample includes households that reported at least one transaction in both the pre- and post-pandemic period (11th week of 2020), where the average age of household heads is 55 or younger, that identify as Black or white, and with a record of purchasing cigarettes in the past four weeks. The outcome is the weekly volume of cigarette purchases (count). The models control for time (year, week in year), and household fixed effects, as well as the main effect of parent status and race/ethnicity-by-parent status. SAC stands for school-aged children (aged 6 to 17) and YC is young children (under 6). | | |

Appendix Table 4. Estimation Results, Other Spending Outcomes

|  | Spending on Cigarettes ($) | | | |  | Spending Share on Cigarettes | | | |  | Spending on Tobacco ($) | | | |
| --- | --- | --- | --- | --- | --- | --- | --- | --- | --- | --- | --- | --- | --- | --- |
|  | (1) | (2) | (3) | (4) |  | (5) | (6) | (7) | (8) |  | (9) | (10) | (11) | (12) |
|  |  |  |  |  |  |  |  |  |  |  |  |  |  |  |
| Pandemic | 2.657*** | 2.633*** | 3.338*** | 3.299*** |  | 0.0106** | 0.00729 | 0.0100* | 0.00998* |  | 2.520*** | 2.460*** | 3.091*** | 3.026*** |
|  | (0.607) | (0.640) | (0.716) | (0.716) |  | (0.00358) | (0.00383) | (0.00439) | (0.00439) |  | (0.634) | (0.672) | (0.759) | (0.759) |
|  |  |  |  |  |  |  |  |  |  |  |  |  |  |  |
| Pandemic x Hispanic |  |  | -1.007 |  |  |  |  | 0.00220 |  |  |  |  | -0.705 |  |
|  |  |  | (1.349) |  |  |  |  | (0.00844) |  |  |  |  | (1.368) |  |
|  |  |  |  |  |  |  |  |  |  |  |  |  |  |  |
| Pandemic x Black |  |  |  | -1.757 |  |  |  |  | -0.0122 |  |  |  |  | -1.355 |
|  |  |  |  | (1.363) |  |  |  |  | (0.00944) |  |  |  |  | (1.386) |
|  |  |  |  |  |  |  |  |  |  |  |  |  |  |  |
| Pandemic x Parents |  | 0.064 | -0.534 | -0.538 |  |  | 0.0102* | 0.00971 | 0.00970 |  |  | 0.175 | -0.363 | -0.367 |
|  |  | (0.826) | (0.958) | (0.958) |  |  | (0.00492) | (0.00583) | (0.00583) |  |  | (0.857) | (1.005) | (1.005) |
|  |  |  |  |  |  |  |  |  |  |  |  |  |  |  |
| Pandemic x Parents x Hispanic |  |  | -1.336 |  |  |  |  | -0.0164 |  |  |  |  | -1.635 |  |
|  |  |  | (2.353) |  |  |  |  | (0.0125) |  |  |  |  | (2.375) |  |
|  |  |  |  |  |  |  |  |  |  |  |  |  |  |  |
| Pandemic x Parents x Black |  |  |  | 2.955 |  |  |  |  | 0.00304 |  |  |  |  | 2.807 |
|  |  |  |  | (2.117) |  |  |  |  | (0.0146) |  |  |  |  | (2.169) |
|  |  |  |  |  |  |  |  |  |  |  |  |  |  |  |
| Observations | 101,284 | 101,284 | 85,946 | 85,862 |  | 101,273 | 101,273 | 85,938 | 85,854 |  | 101,284 | 101,284 | 85,946 | 85,862 |
| Number of households | 3,317 | 3,317 | 2,765 | 2,742 |  | 3,317 | 3,317 | 2,765 | 2,742 |  | 3,317 | 3,317 | 2,765 | 2,742 |
|  |  |  |  |  |  |  |  |  |  |  |  |  |  |  |
| **SOURCE** Authors' analysis of data from the NielsenIQ Homescan Consumer Panel, 2019-2020. **NOTES** *** p<0.001, ** p<0.01, * p<0.05. Household-clustered standard errors are in parentheses. The analytic sample, the sample restrictions, the models are the same as those used in Appendix Table 2. Controls from the baseline model are included. The outcomes variables are total weekly spending on cigarettes ((1)-(4)); the total spending on cigarettes is divided by the total weekly spending in the households ((5)-(8)); and total weekly spending on all tobacco products, including cigars, chewing tobacco, and e-cigarettes ((9)-(12)). | | | | | | | | | | | | | | |

Appendix Table 5. Estimation Results, Other Outcomes

|  | Number of Cigarette per Adult (ct) | | | |  | Price per Pack ($) | | | |  | Buying nicotine replacement therapy | | | |
| --- | --- | --- | --- | --- | --- | --- | --- | --- | --- | --- | --- | --- | --- | --- |
|  | (1) | (2) | (3) | (4) |  | (5) | (6) | (7) | (8) |  | (9) | (10) | (11) | (12) |
|  |  |  |  |  |  |  |  |  |  |  |  |  |  |  |
| Pandemic | 4.612** | 4.786** | 5.748** | 5.475** |  | -0.162*** | -0.176*** | -0.129* | -0.124* |  | -0.000 | -0.001 | -0.001 | -0.001 |
|  | (1.485) | (1.518) | (1.783) | (1.786) |  | (0.047) | (0.053) | (0.056) | (0.055) |  | (0.001) | (0.001) | (0.001) | (0.001) |
|  |  |  |  |  |  |  |  |  |  |  |  |  |  |  |
| Pandemic x Hispanic |  |  | 0.0333 |  |  |  |  | -0.210 |  |  |  |  | 0.002 |  |
|  |  |  | (2.230) |  |  |  |  | (0.147) |  |  |  |  | (0.001) |  |
|  |  |  |  |  |  |  |  |  |  |  |  |  |  |  |
| Pandemic x Black |  |  |  | -2.905 |  |  |  |  | 0.054 |  |  |  |  | 0.002* |
|  |  |  |  | (2.935) |  |  |  |  | (0.109) |  |  |  |  | (0.001) |
|  |  |  |  |  |  |  |  |  |  |  |  |  |  |  |
| Pandemic x Parents |  | -0.563 | -1.533 | -1.530 |  |  | 0.042 | -0.015 | -0.016 |  |  | 0.001* | 0.002* | 0.002* |
|  |  | (1.639) | (1.993) | (1.994) |  |  | (0.058) | (0.067) | (0.067) |  |  | (0.001) | (0.001) | (0.001) |
|  |  |  |  |  |  |  |  |  |  |  |  |  |  |  |
| Pandemic x Parents x Hispanic |  |  | -2.830 |  |  |  |  | 0.336 |  |  |  |  | -0.002 |  |
|  |  |  | (4.034) |  |  |  |  | (0.173) |  |  |  |  | (0.001) |  |
|  |  |  |  |  |  |  |  |  |  |  |  |  |  |  |
| Pandemic x Parents x Black |  |  |  | 6.841 |  |  |  |  | -0.103 |  |  |  |  | -0.003* |
|  |  |  |  | (4.445) |  |  |  |  | (0.180) |  |  |  |  | (0.001) |
|  |  |  |  |  |  |  |  |  |  |  |  |  |  |  |
| Observations | 101,284 | 101,284 | 85,946 | 85,862 |  | 56,013 | 56,013 | 47,887 | 48,081 |  | 254,826 | 254,826 | 211,231 | 210,569 |
| Number of households | 3,317 | 3,317 | 2,765 | 2,742 |  | 2,633 | 2,633 | 2,215 | 2,201 |  | 3,369 | 3,369 | 2,804 | 2,786 |
|  |  |  |  |  |  |  |  |  |  |  |  |  |  |  |
| **SOURCE** Authors' analysis of data from the NielsenIQ Homescan Consumer Panel, 2019-2020. **NOTES** *** p<0.001, ** p<0.01, * p<0.05. Household-clustered standard errors are in parentheses. The analytic sample, the sample restrictions, the models are the same as those used in Appendix Table 2. Controls from the baseline model are included. The outcomes variables are total weekly cigarettes per adult in the household (as measured in late 2019) ((1)-(4)); the price paid per pack of cigarettes ((5)-(8)); and an indicator for having spent any money on nicotine replacement therapy (NRT) products ((9)-(12)). For the model on nicotine replacement therapy, the sample is restricted to households that purchased cigarettes at least once during the sample period. | | | | | | | | | | | | | | |

Appendix Table 6. Cigarette Purchase Behavior, April-December 2019 versus April-December 2020

|  |  | Mean | |  |  |
| --- | --- | --- | --- | --- | --- |
|  |  | Apr-Dec 2019 | Apr-Dec 2020 | Difference | t-stat |
|  |  |  |  |  |  |
| All | Buying | 0.094 | 0.104 | *** | 3.59 |
|  | Number (ct) | 1,142.3 | 1,288.6 | * | 2.04 |
|  | Spending ($) | 347.9 | 408.3 | ** | 2.77 |
|  |  |  |  |  |  |
| Parents | Buying | 0.075 | 0.081 |  | 1.35 |
|  | Number (ct) | 872.93 | 1,116.22 | * | 2.35 |
|  | Spending ($) | 267.22 | 356.50 | ** | 2.93 |
|  |  |  |  |  |  |
| Black Parents | Buying | 0.081 | 0.082 |  | 0.09 |
|  | Number (ct) | 612.16 | 788.57 |  | 0.84 |
|  | Spending ($) | 214.13 | 257.18 |  | 0.62 |
|  |  |  |  |  |  |
| Hispanic Parents | Buying | 0.086 | 0.074 |  | -1.04 |
|  | Number (ct) | 541.33 | 540.19 |  | -0.01 |
|  | Spending ($) | 182.35 | 196.25 |  | 0.30 |
|  |  |  |  |  |  |
| **SOURCE** Authors' analysis of data from the NielsenIQ Homescan Consumer Panel, 2019-2020. **NOTES** *** p<0.001, ** p<0.01, * p<0.05. Analytical sample described in Appendix Table 1. *Buying* indicates any spending on cigarettes between April and December 2019, or between April and December 2020. The number and spending are the sum of purchased cigarettes and spending on cigarettes during each period and are conditional on households who made at least one cigarette purchase during each respective period. | | | | | |

Appendix Table 7. Estimation Results, Purchase Outcomes, Low Educational Attainment

|  | Buying Cigarettes | | | | |  | Number of Cigarettes (ct) | | | | |
| --- | --- | --- | --- | --- | --- | --- | --- | --- | --- | --- | --- |
|  | Baseline | State SxY FE |  | Baseline | State SxY FE |  | Baseline | State SxY FE |  | Baseline | State SxY FE |
|  | (1) | (2) |  | (3) | (4) |  | (5) | (6) |  | (7) | (8) |
|  |  |  |  |  |  |  |  |  |  |  |  |
| Pandemic | 0.00066 | 0.00068 |  | 0.00070 | 0.00072 |  | 11.246*** | 11.151*** |  | 11.235*** | 11.194*** |
|  | (0.00078) | (0.00078) |  | (0.00078) | (0.00079) |  | (2.727) | (2.723) |  | (2.731) | (2.722) |
|  |  |  |  |  |  |  |  |  |  |  |  |
| Pandemic x Low Education | -0.00564* | -0.00561* |  | -0.00567* | -0.00563* |  | 0.411 | 1.384 |  | 0.385 | 1.350 |
|  | (0.00259) | (0.00259) |  | (0.00259) | (0.00259) |  | (3.223) | (3.119) |  | (3.222) | (3.120) |
|  |  |  |  |  |  |  |  |  |  |  |  |
| Pandemic x Parents | 0.00001 | -0.00003 |  |  |  |  | 0.258 | 0.546 |  |  |  |
|  | (0.00078) | (0.00078) |  |  |  |  | (3.120) | (2.883) |  |  |  |
|  |  |  |  |  |  |  |  |  |  |  |  |
| Pandemic x Parents x Low Education | 0.00121 | 0.00124 |  |  |  |  | -3.009 | -7.398 |  |  |  |
|  | (0.00375) | (0.00374) |  |  |  |  | (6.346) | (6.033) |  |  |  |
|  |  |  |  |  |  |  |  |  |  |  |  |
| Pandemic x Parents x SAC |  |  |  | -0.00014 | -0.00014 |  |  |  |  | 0.287 | 0.294 |
|  |  |  |  | (0.00080) | (0.00081) |  |  |  |  | (3.283) | (3.024) |
|  |  |  |  |  |  |  |  |  |  |  |  |
| Pandemic x Parents x YC |  |  |  | 0.00042 | 0.00007 |  |  |  |  | 0.998 | 2.389 |
|  |  |  |  | (0.00149) | (0.00147) |  |  |  |  | (4.625) | (4.727) |
|  |  |  |  |  |  |  |  |  |  |  |  |
| Pandemic x Low Education x SAC |  |  |  | -0.00029 | -0.00030 |  |  |  |  | -3.528 | -7.360 |
|  |  |  |  | (0.00392) | (0.00391) |  |  |  |  | (6.725) | (6.369) |
|  |  |  |  |  |  |  |  |  |  |  |  |
| Pandemic x Low Education x YC |  |  |  | 0.01691** | 0.01723** |  |  |  |  | 6.683 | -1.843 |
|  |  |  |  | (0.00604) | (0.00606) |  |  |  |  | (11.362) | (12.344) |
|  |  |  |  |  |  |  |  |  |  |  |  |
| Observations | 1,911,675 | 1,911,675 |  | 1,911,675 | 1,911,675 |  | 101,284 | 101,284 |  | 101,284 | 101,284 |
| Number of households | 24,795 | 24,795 |  | 24,795 | 24,795 |  | 3,317 | 3,317 |  | 3,317 | 3,317 |
|  |  |  |  |  |  |  |  |  |  |  |  |
| Means |  |  |  |  |  |  |  |  |  |  |  |
| (pre-pandemic) | 0.03 | | | | |  | 63.25 | | | | |
| (pre-pandemic, low education) | 0.08 | | | | |  | 77.22 | | | | |
| (pre-pandemic, parents) | 0.02 | | | | |  | 53.42 | | | | |
| (pre-pandemic, parents, low education) | 0.05 | | | | |  | 58.32 | | | | |
|  |  |  |  |  |  |  |  |  |  |  |  |
| **SOURCE** Authors' analysis of data from the NielsenIQ Homescan Consumer Panel, 2019-2020. **NOTES** *** p<0.001, ** p<0.01, * p<0.05. Household-clustered standard errors are in parentheses. Analytical sample includes households that reported at least one transaction in both the pre- and post-pandemic period (11th week of 2020), and where the average age of household heads is 55 or younger. The outcome variable is a binary indicator of whether a household reported purchasing any cigarettes in a week ((1)-(4)), and the number of weekly cigarettes purchased conditional on having bought any in the previous four weeks ((5)-(8)). Columns (1), (3), (5), and (7) represent the baseline model, which controls time (year, week in year), and household fixed effects. Columns (2), (4), (6), and (8) include household characteristics, state, state-year fixed effect in addition to the baseline model specifications. All models also include controls for the main effect of parent status and race/ethnicity-by-parent status. *Low education* is defined as households in which the highest education level among heads is a high school diploma or less. *SAC* stands for school-aged children (aged 6 to 17) and *YC* is young children (under 6). Pre-pandemic means of the dependent variables are provided below DD and DDD coefficients. | | | | | | | | | | | |

Appendix Table 8. Estimation Results, Cigarette Purchase, Heads Between 55 and 75 Years Old

|  | Buying Cigarettes | |  | Number of Cigarettes (ct) | |
| --- | --- | --- | --- | --- | --- |
|  | Baseline | State SxY FE |  | Baseline | State SxY FE |
|  | (1) | (2) |  | (3) | (4) |
|  |  |  |  |  |  |
| Pandemic | 0.00069 | 0.00069 |  | 13.486*** | 13.616*** |
|  | (0.00052) | (0.00052) |  | (2.739) | (2.726) |
|  |  |  |  |  |  |
| Observations | 2,548,887 | 2,548,887 |  | 136,395 | 136,395 |
| Number of households | 30,142 | 30,142 |  | 3,700 | 3,700 |
|  |  |  |  |  |  |
| Mean (pre-pandemic) | 0.033 | |  | 102.75 | |
|  |  |  |  |  |  |
| **SOURCE** Authors' analysis of data from the NielsenIQ Homescan Consumer Panel, 2019-2020. **NOTES** *** p<0.001, ** p<0.01, * p<0.05. Household-clustered standard errors are in parentheses. Analytical sample includes households that reported at least one transaction in both the pre- and post-pandemic period (11th week of 2020), and where the average age of household heads is between 55 and 75. The outcome variable in columns (1) and (2) is a binary indicator of whether a household reported purchasing any cigarettes in a week. The outcome in columns (3) and (4) is the weekly number of cigarettes purchased (count), conditional on having purchased cigarettes in the past four weeks. Columns (1) and (3) represent the baseline model, which controls time (year, week-in-year), and household fixed effects. Columns (2) and (4) include household characteristics, state, state-year fixed effect in addition to the baseline model specifications. | | | | | |

Appendix Figure 2. Effects of the pandemic on cigarettes spending, 2019-2020

| 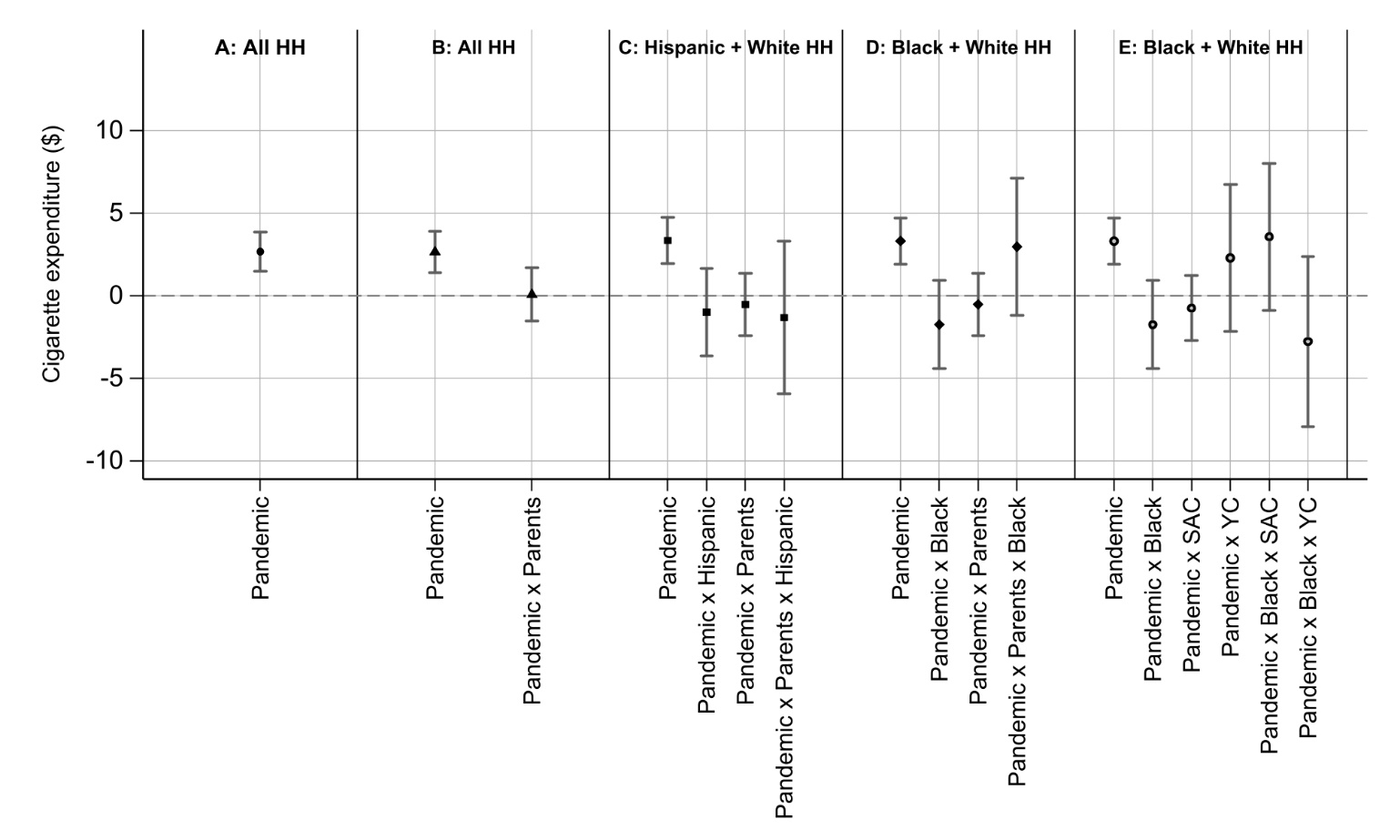 |
| --- |
| **SOURCE** Authors' analysis of data from the NielsenIQ Homescan Consumer Panel, 2019-2020. **NOTES** The outcome is the weekly spending on cigarettes ($). The sample is restricted to households with a record of purchasing cigarettes in the past four weeks. The models and coefficients for each panel are described in the note of Figure 1. |
